# Exploring the nature of stigmatising beliefs about depression and help-seeking: Implications for reducing stigma

**DOI:** 10.1186/1471-2458-9-61

**Published:** 2009-02-20

**Authors:** Lisa J Barney, Kathleen M Griffiths, Helen Christensen, Anthony F Jorm

**Affiliations:** 1 Centre for Mental Health Research, The Australian National University, Canberra, ACT, 0200, Australia; 2 ORYGEN Research Centre, Department of Psychiatry, University of Melbourne, Locked Bag 10, Parkville, Victoria 3052, Australia

## Abstract

**Background:**

In-depth and structured evaluation of the stigma associated with depression has been lacking. This study aimed to inform the design of interventions to reduce stigma by systematically investigating community perceptions of beliefs about depression according to theorised dimensional components of stigma.

**Methods:**

Focus group discussions were held with a total of 23 adults with personal experience of depression. The discussions were taped, transcribed and thematically analysed.

**Results:**

Participants typically reported experiencing considerable stigma, particularly that others believe depressed people are responsible for their own condition, are undesirable to be around, and may be a threat. Participants expressed particular concerns about help-seeking in the workplace and from mental health professionals.

**Conclusion:**

Findings indicate that interventions to reduce the stigma of depression should target attributions of blame; reduce avoidance of depressed people; label depression as a 'health condition' rather than 'mental illness'; and improve responses of help-sources (i.e. via informing professionals of client fears).

## Background

Stigmatising beliefs about depression, whether actual or perceived, can have a substantial impact on people with depression. Perceptions that others may respond negatively may lead to help-seeking avoidance [1-4], somatisation and misdiagnosis [5-7], and treatment discontinuation [8]. Clearly, if our goal is early intervention and depression treatment, we need to address stigma of depression. However, effective intervention strategies require a thorough understanding of the nature of existing beliefs, and our current understanding of depression stigma is limited.

It appears that negative views about depression and help-seeking are common in the general public. Many people believe those with depression are hard to talk to, unpredictable and threatening to others [9-11], and consider medical treatment for depression to be unnecessary [12]. Other research has found that people seeking psychological counselling are viewed as being defensive, awkward, insecure, sad, cold and unsociable [13,14], and that people with depression who seek help from mental health professionals are seen as more unstable than those who do not [15].

Accordingly, it is not surprising that many people believe others hold negative views about depression and help-seeking. Griffiths et al. (2006) found that 53% of their community sample believed that most people would think depression is a sign of personal weakness, and 38% believed others think people with depression are dangerous. People with depression have reported concerns that others would see them as being lazy, weak, incapable of coping with life, and inferior [16-18]. Fears of stigma have also been reported with respect to disclosing depression to informal sources of help such as family, friends, and work colleagues, and in relation to help-seeking from professionals [e.g. [1,3,17,19,20]].

It is apparent that stigma about depression is a problem. However, there has been little research to date about stigma of depression based on a comprehensive framework of the key elements of stigma. In-depth investigation is needed because although depression constitutes a mental illness, stigmatising beliefs about depression are not the same as those held about other mental illnesses [see [21-25]].

A six-dimensional, structured approach proposed by Jones et al [26] has been used to examine stigma associated with health conditions: *Concealability *refers to whether or not the condition is obvious to others, and the extent to which its visibility is controllable; *course *means the typical pattern of change, and outcome of the condition; *disruptiveness *relates to the extent to which the condition hinders interaction and communication; *aesthetic qualities *refers to the extent to which it makes the sufferer repellent, ugly, or upsetting; *origin *relates to the circumstances under which the condition originated, including attributions of responsibility for the condition; and *peril *concerns the likelihood, imminence and severity of danger to others. A condition is generally considered less stigmatising if it is easily concealed, minor and of short duration with a good outcome, non-disruptive, non-repellent, not the fault of the sufferer, and not dangerous to others [26].

One method for investigating the nature of public stigmatising attitudes is by means of a representative survey of the community. However, in practice, this method may be problematic because people who are uninterested may decline to participate in the study [27], and those participating may be reluctant to disclose their stigmatising beliefs, believing them to be socially unacceptable [28]. An alternative approach to considering the presence of stigma and its themes is to investigate the views and experiences of people who have experienced mental illness.

The current study qualitatively examined the perspectives of people with a history of depression in relation to the dimensions of stigma and help-seeking for depression. Jones et al's (1984) six dimensions were used as potential themes of stigma. However, in order to more accurately reflect the issues concerning depression, several of the dimensions were adapted. *Disruptiveness *was expanded to include problems other than interpersonal difficulties and labelled *difficulties*. Given that depression is not a disfiguring condition, *aesthetic qualities *was adapted to the concept of *repellence*, which encompasses other attributes of depressed people considered repellent (e.g. demeanour). *Peril *was renamed *threat *to represent a broader theme, and *origin *renamed *responsibility*. The aim of the study was to determine the emphasis and concordance of these dimensions of stigma with reported perspectives, and whether additional themes are needed to describe the nature of stigma associated with depression.

## Methods

Focus group methodology was chosen because it is considered to be an effective technique for exploring people's views about particular issues and examining why they hold such views [29] and for obtaining in-depth knowledge about attitudes, particularly in relation to public health [30]. Group discussions are believed to be advantageous over individual interviews because the former generate a greater diversity of responses; may encourage people to speak out about sensitive topics; and do not involve as much interaction between participants and moderators thereby decreasing the influence of the researcher in the process and allowing participants' views to be more prominent [30,31]. Groups were kept small to maximise the depth of content elicited, and the decision regarding the number of required groups was reserved until findings indicated that 'saturation' of views (when additional information does not increase understanding) [32] was achieved.

Participants were informed that published material would not include identifying information, and written consent was obtained prior to the discussions. Participants were offered travel reimbursement to attend the discussions. The Australian National University Human Research Ethics Committee approved the study.

### Sampling

Participants were adult members of the community recruited from a depression support group in Canberra or through a newspaper advertisement, who reported past or present major depression. People were included if they had sought professional help and been diagnosed with depression, or had not sought help but on questioning were found to currently meet the diagnostic criteria for Major Depression according to the DSM-IV [33] or would have met such criteria in the past. Four groups were formed.

### Data collection

Each focus group discussion was of approximately two hours duration, and was semi-structured, based around questions in a research guide developed for the study, yet allowing topics to be pursued as they arose. Participants were asked what they thought other people believed about depression and how others responded to people with depression (i.e. "Do you think other people believe depressed people are dangerous?" and "Were there any negative consequences of telling others you were depressed?"). Probing questions were used to follow-up on the primary questions in the research guide for the purpose of extracting more information. The group moderator (LB) had a psychology degree, was experienced in dealing with adult groups, and had training and experience in counselling. Discussions were audiotaped and transcribed verbatim. Saturation of views appeared to be reached after four group discussions.

### Analysis

Thematic analysis of the transcriptions was carried out with the assistance of QDA Miner v1.0 [34], a qualitative data analysis software package used for coding, annotating, retrieving and reviewing textual data. Analysis was based on the approaches of *Meaning categorization *and *Meaning interpretation *[see [35]], whereby categories taken from theory and developed in advance are used to code and allow a deeper interpretation of participant responses. All units of data were examined for themes relating to the dimensions of stigma, and pertaining to stigma associated with help-seeking for depression. Analysis also allowed for recognition of other emergent themes: Data were also examined for other facets of stigma not specified in Jones and colleagues' typology using the data-driven *inductive *approach to thematic analysis [refer to [36]], where data are examined for codes and patterns, and themes are allowed to emerge from the data (in contrast to the predetermination of themes by theory or prior research).

All themes were then extracted and examined in more detail, interpretation of which involved searching the data for concepts, associations and explanations. The emphasis on stigma associated with each dimension was rated from weak to strong according to the number of participants who endorsed the themes, the duration of discussions related to the themes, and the apparent intensity of feeling associated with the themes.

## Results

### Sample characteristics

The participants comprised 14 females and nine males, and ranged in age from 22 to 78 years (*Mean *= 48.9). Demographic details are provided in Table 1.

**Table 1:** Demographic profile of participants (N = 23)

Variables	N	%
Gender		
Male	9	39.1
Female	14	60.9
		
Age (years)		
≤ 25 yrs	1	4.3
26–35 yrs	4	17.4
36–45 yrs	5	21.7
46–55 yrs	5	21.7
56–65 yrs	5	21.7
≥ 66 yrs	3	13.0
		
Education		
Primary	0	0
4 years secondary	4	17.4
6 years secondary	3	13.0
Certificate/diploma	7	30.4
Bachelors degree	9	39.1

### Themes

Findings are reported according to themes corresponding to the six dimensions and to help-seeking. Although investigation of the material was not restricted to these dimensions, no additional dimensions of stigma were evident.

#### Perceived stigma regarding dimensions of stigma

##### Concealability

The commonly expressed view by participants was that depression, by its nature, is not usually obvious to other people. The predominant response was that the concealability of depression constituted a benefit, in that it might be deliberately concealed if desired. Many participants said they were able to successfully conceal it to avoid negative responses and consequences:

"I don't think anyone I'd ever met would say that I wasn't a happy optimistic person. I'd never, never bring anything bad to work, never sound unhappy, nothing, and so it was all very well hidden, and I still hide it now."

"I've become very good [at hiding my depression], in fact, I'm sure people don't realise what's going on."

On the other hand, others considered the hidden nature of depression to be a problem because it hampers its recognition and treatment, as demonstrated in these comments:

"Because [depression] is largely invisible ... your non-participation is given other reasons, some of which might not be useful, so it's a handicap and the invisibility of it is a problem."

"Everyone says 'but you look so well', you know, 'you look great', and 'there's nothing wrong, don't be silly', ... maybe if I got help earlier, if someone had identified it and treated it more seriously, things would have been better."

Whether its invisibility was considered to be good or bad, there was agreement that depression is not usually apparent to others. Lower visibility is generally associated with lesser stigma (Jones, 1984), therefore, it appears that for depression the dimension of *concealability *is not one that attracts stigma.

##### Course

Misunderstanding about depression as an illness was a primary theme amongst the groups. Overwhelmingly, participants believed that often others see depression as mere sadness or low mood, and expect it to resolve by itself in the short term. There was very strong agreement with the following statement which refers to the tendency of others to underrate depression:

"Another thing I've heard from people about my depression is they say 'oh, we all get sad'."

Interestingly, there was a spectrum of how participants wanted the illness perceived. Some were fearful that people might see them as having a mental illness, and believed that the association of depression with the label 'mental illness' caused a problem:

"You wear it from the day you are diagnosed with a 'mental illness', and that's the stigma. Depression on its own, well, it's the new fashion to say you've got depression, but you say you've got a mental illness – they look at you and they expect you to be a dribbling idiot."

"If you say you suffer from 'a mental illness', they take a step back. It's nearly as bad as saying 'I've got AIDS' – they're frightened."

In contrast, others *wanted *depression to be recognised as a mental illness, and therefore taken seriously:

"...they wouldn't believe it was actually a mental illness – which it is of course, it's one of the most damaging and cruel reactions to depression, ... this under-rating..."

Although underestimation of the course of depression may itself not be desirable, beliefs of lesser severity are generally associated with lesser stigma (Jones et al, 1984). Therefore, perceptions that depression is not considered by others to be a serious illness may be seen to suggest that depression is not highly stigmatised in relation to the dimension of *course*.

##### Difficulties

The majority of participants felt that others do not understand – and that they underestimate – the extent of difficulties associated with depression.

"I think depression affects your whole life hugely, and I think most of the time people don't really understand that."

However, other reports indicate that *over*estimation of difficulties may also be a problem. These concerns were mainly apparent in relation to the workplace setting, whereby participants thought that others believe people with depression *do *experience serious difficulties with work demands:

"Mostly with work I haven't told people, and you feel as though if you do tell them they're just going to think you're mentally... you know, incompetent, a bit strange, that's how I feel they're going to react, that you may not be able to do your work."

The strong emphasis on perceptions of underestimation of difficulties suggests that the dimension of difficulties is not typically associated with stigma. However, perceptions related to the workplace do suggest that some stigma is associated with this aspect. Both of these types of views appear to pose concerns for people with depression, indicating the need for careful consideration of beliefs about this dimension.

##### Repellence

Repellence in relation to depression is conceived as being an intricate concept that extends beyond features of a visually aesthetic type. Many participants reported that others do not want to be around depressed people because of the inherent negativity of the depressive condition:

*"People don't sort of want your company when you're not in good spirits*."

Repellence may also be attributable to the label of depression itself. There was a commonly-held view that others are uncomfortable with being presented with a diagnosis of depression, as shown in this remark:

*"I could use the word 'depression'... and you can see it in their face... it's the same sort of response... they're automatically sorry they've asked the question*."

Reports suggest that discomfort may occur in others because they do not know what to do or say:

"... they just don't know how to handle it... it's not their fault, you can't blame them ..."

Repellence may also be something which does not necessarily exist at first, but which can develop when depression persists. Some participants expressed the view that others often do try to help at first, but that it lessens over time:

*"I think generally people who deal with people who are depressed ... don't feel ill-willed towards them to any great degree, but I think over a period of time it may change*."

Responses indicate that there is a moderately-high emphasis on stigma associated with the dimension of *repellence*.

##### Responsibility

Perceptions that people are believed to be responsible to some extent for their own depression were pervasive. With very few exceptions, participants felt acutely that others blame them for their depression, believing that they can and should do something to get over it, or in other words "snap out of it":

*"Most people seem to think depression is... something that is within your character to control*."

Notably, people with depression may believe that blame is inevitable if they have no good reason for their depression:

*"I think if there was a reason you could say definitely caused it, you know, the big car accident or the big funeral, people would go 'oh, I understand', but if you can just sit there and say 'look, I really don't know where it all started', it would be like ... you've been a bit nutty all your life, there's something wrong with you.*"

The number and strength of responses concerning perceptions of blame indicate that the dimension of *responsibility *is one that attracts high levels of concern in relation to depression.

##### Threat

While the majority of participants did not seem worried that others might see them as a threat in some way, some concerns were evident. One participant reported that a relative no longer wanted her to baby-sit, and other participants referred to a detrimental effect of the association of depression with a 'mental illness' label:

"... *there's a perception that anyone with a mental illness is dangerous*."

Although perceptions regarding the dimension of *threat *were not stressed by most participants, the strength of existing comments suggested that for some people this dimension attracted considerable fears of stigmatising responses from others.

#### Perceived stigma regarding help-seeking for depression

Almost without exception, participants said they had restricted disclosing their depression because they believed other people would react negatively to them if they knew about their depression. In some cases, fears of negative responses were based on anticipation only, but in other cases, expectations were based upon actual experiences of help-seeking. Reluctance to disclose depression applied to a variety of help-sources.

##### Informal help-seeking

Participants reported concerns about responses from informal sources of support arising from disclosure of depression itself:

"....*because what was happening to me [depression]... is usually misinterpreted in a negative way, either through presuming someone to be stupid or presuming someone to be weak, ... it certainly discourages people to talk to friends about it or anyone*."

Expectations of negative responses from informal sources of help were common. Quite a number of participants said they had not told friends or members of their family about their depression, and men seemed particularly reluctant to tell their friends:

"Telling people is sort of showing your weakness, your underside, and they'll think less of you because you're weak and you can't cope with life."

"I think you grow up with it – men don't cry ... it's the social group that does it, I think they always have and probably are still doing it – 'don't be a sissy'."

There seemed to be even greater reservations about telling work acquaintances:

*"You're afraid of ridicule, I found that... even though you may be close to someone [at work], you can't really tell them really personal stuff because you're scared they're going to 'take the Mickey' or go and tell someone about it*."

Even the possibility that other people may respond with sympathy or tolerance was not always seen as desirable. Many participants expected that if they disclosed their depression to others they were likely to be treated differently, and it seemed that underlying this were concerns about being seen in a lesser way:

"That people would know and would just take account of the fact that you might have had a bad day ... for me, I would find that very condescending to be treated differently."

"You think they'll never look at me the way they used to, they'll always look at me as though I've got problems ... you don't want them to have these bad opinions of you."

Expectations of stigmatising responses from various sources of informal help were common. Additionally, concerns about other responses – which were not necessarily stigmatising but seen as being undesirable nonetheless – were also apparent. Thus, it appears that concerns about responses from informal sources are a major and somewhat complex problem.

##### Professional help-seeking

Concerns about help-seeking from professionals were also predominant, with the vast majority of participants reporting some concerns about the consequences of such help-seeking. Fears about the responses of *other people *in relation to professional help-seeking were common. Participants were afraid that others may respond badly, although these concerns varied according to the type of professional help-source. There was little stigma evident regarding seeking help from general practitioners, perhaps because sufferers could camouflage the reason for help-seeking, but there was considerable unease about other people's responses towards seeking help from mental health professionals. Psychiatrists specifically were mentioned by many of the participants. Such concerns were especially common in males, as help-seekers and in response to other people's help-seeking:

"For many years I put off getting help, mainly because my husband didn't want me to. He thought that it put some stigma onto him if he had a wife who had depression and who needed psychiatric help."

Additionally, many participants did not want other people to know that they were taking antidepressants. One woman said she had felt anxious giving her prescription over in the pharmacy in case others around her heard the pharmacist refer to her medication. The following quote illustrates a commonly reported scenario where sufferers somewhat reluctantly take antidepressants, and do not disclose their use to other people:

"I don't like taking [antidepressants] but I accept that I do need to take them, and no, I don't tell anybody."

Participants also reported fearing negative responses from professionals themselves. Some concerns were evident in relation to *expectations *of stigmatising reactions from general practitioners, and in those participants who had sought such help, *experiences *were mixed. While some had received good management, others reported bad experiences with their doctors and felt upset about the way they had been treated. Help-seeking from mental health professionals was associated with stronger reports of stigmatising responses. Expectations of negative responses from psychiatrists were clearly evident, with these comments receiving strong agreement:

"I'm finding it very difficult to go back to see the same psychiatrist again. On the first visit he had a pretty condescending attitude – without saying as much he kind of indicated 'you've got to pull yourself together'."

"You're treated as if you've done something or something's happened for you to get this... and this is from trained psychiatrists specialising in mental illness."

Participant reports indicate that help-seeking from professionals is associated with substantial concerns about stigmatising responses from other people in relation to the help-seeking and from professionals themselves.

## Discussion

The current findings suggest that stigma is a substantial concern for people with personal experience of depression, with the vast majority of participants reporting perceptions of stigmatising beliefs or responses from others. Themes given a lot of emphasis involved beliefs that other people think those with depression are responsible for their condition, and find people with depression repellent. Beliefs that others see those with depression as being a threat existed although they were less widespread. Strongly evident were beliefs that other people are often unaware when a person is depressed, and underestimate the severity and duration of depression and the extent of the difficulties it causes. In relation to help-seeking, concerns about disclosing depression to informal sources and professionals were commonly expressed. Below we discuss separately stigma regarding the dimensions and stigma associated with help-seeking.

### Stigma regarding dimensions

The emphasis given to the dimension of *responsibility *supports other qualitative studies that have found perceptions of blame from other people are strong concerns of people with depression [e.g. [1,17,37]]. Indeed, according to the finding that 43% of Australian adults believe a weak character is a likely cause of depression [38], such concerns may be accurate. The theme of blame may be particularly important to people with depression because they suspect that support from others is less likely if people believe them to be responsible for their condition. The possibility that others would be less supportive in such circumstances is consistent with research findings that attributions of greater controllability are linked to a lesser willingness to help [39,40]. Findings in the present study also suggest that provision of a reason (justification) for depression may play an important role in reducing blame. The notion that being able to identify a reason for depression helps to explain one's behaviour to others and so makes it more acceptable is also evident in findings from another qualitative study [see [41]]. It is apparent that the issue of blame for depression is of primary importance, and that the provision of an accurate and understandable causal explanation for depression might be beneficial.

The present findings indicate that stigma concerning the dimension of *repellence *is also a major concern for those with depression. These concerns relate to perceptions that others do not find them pleasant to be around and either have avoided or will eventually give up on them. This finding is consistent with quantitative research that found 36% of people in the Australian community believe most people think it is best to avoid those with depression [22]. It is possible that avoidant responses are a consequence of beliefs about the behaviours of people with depression, for example, that people with depression are hard to talk to [9]. Alternatively, the reports by participants in the current study that others withdraw upon being told of the diagnosis of depression suggest that the label of depression itself may be sufficient to cause withdrawal. Both explanations are consistent with the suggestion that the desire to avoid undesirable attributes may explain the preference for social distance from people with mental illness [10]. As indicated in the present study, it is possible that preferences for avoidance are due to discomfort arising from not knowing what to say or do to assist someone with depression. If this is the case, educating the public about how to support a depressed person may be helpful in reducing negative responses.

The effect of labelling depression as a 'mental illness' appears to be controversial amongst people with depression. Findings here showed that some participants wanted depression to be recognised as a mental illness to provide it with the status of a 'real' illness. However, other participants were concerned that others believe someone labelled as having a mental illness is dangerous. As to whether labelling depression as a mental illness does increase beliefs about dangerousness, research indicates mixed results: Some studies have concluded that labelling has little effect on attitudes towards those with depression [42], whereas others have reported that people who label depression as a mental illness express a desire for greater social distance [43]. Whether or not beliefs about threat from people with depression are due to labeling, concerns do appear to exist amongst the general public. In an Australian community study, Griffiths et al. [22] found that 42% believe people with depression are unpredictable, and 12% believe they are dangerous. Such levels of concern would support the perceptions of some participants in the current study, and indicate that stigmatising beliefs concerning the dimension of *threat *can be a substantial problem. In addressing this problem, referring to depression as a 'mental illness' may be unhelpful, and it may be preferable to use a term such as 'health condition'. These conclusions also indicate there is a need to increase public knowledge about depression in relation to the issue of threat.

Perceptions concerning the dimensions of *concealability*, *course*, and *difficulties *and their impact need careful consideration. Certainly, depression is not highly visible, and despite indications that many people with depression have recurring episodes [44], the public tends to view depression as being short-term and treatable, and expects that those with depression will recover fully and quickly without relapse [9,23,45]. Although the literature on stigma tends to emphasise the benefits of invisibility and perceptions of lesser seriousness [e.g. [26,46]], dimensions are complex and may not necessarily operate in the anticipated way [26]. For example, although lack of visibility and beliefs of lack of seriousness would generally be seen as representing lower stigma (see Jones, 1984), the current findings indicate that in the context of depression such views may also be seen as a problem. This may be because if people with depression are not fully functioning or do not recover as quickly or fully as expected, responsibility (blame) for continuing depression may be placed upon them. Certainly, people with depression commonly express the desire for others to take depression more seriously, and understand that depression is a severe and long-term illness and that people with depression can experience extreme difficulties. Greater knowledge itself, however, may not reduce stigma, as it has been found that professionals are more negative than the general public about the long-term outcomes for people with depression [25]. Similarly, and as implied in the Jones et al. (1984) framework, a better understanding by the public about the course of depression and the difficulties faced by sufferers may in fact act to increase stigmatising responses regarding these dimensions. Whilst education about the course and difficulties of depression is needed, it is apparent that careful consideration of the delicate balance in the provision of details is required.

There also appears to be an intricacy in relation to perceptions regarding *course *and *difficulties*, such that there are somewhat opposing or contradictory views about expected responses from other people. The current data suggest that those with depression commonly believe that others *under*estimate the difficulties faced by sufferers. However, other reports by participants of anticipated or actual discrimination in the workplace also indicate beliefs that other people (especially employers) *over*estimate their difficulties and hold the view that people with depression are not capable of performing work duties. Indeed, expectations that others may consider them incapable of good work performance are substantiated by findings that concerns about workplace capabilities of people with past or present depression are common amongst employers as well as the Australian public [see [22,47,48]]. It is apparent that there is a need to deal with beliefs about these dimensions, particularly in the workplace, and that we need to further verify the intricacies and address them specifically in education programs.

### Stigma regarding help-seeking for depression

The view that confiding in family, friends and work colleagues would result in ridicule or being viewed less favourably features very strongly in the current findings. Not surprisingly, fears of this type may inhibit help-seeking from informal sources [see [17]]. These fears are particularly common with respect to work environments, possibly due to a concern that help-seeking at work may affect job opportunities. As indicated in the findings, the added difficulty is that people with depression may also find less negative responses such as sympathy to be undesirable. Wahl [49] asserted that people with a mental illness may fear being treated differently, even with care and support, because it can be perceived as reflecting *over*concern and underlying views of incompetence. It might be anticipated that sympathetic responses would be seen as being particularly undesirable in the workplace where competency is a highly valued attribute, and fears of such responses may play a key role in reluctance to seek support in work environments.

Concerns about negative responses from friends and family regarding help-seeking from professionals and the use of antidepressants are also strongly evident in the current research. Certainly, there is evidence that the general public rejects the use of anti-depressants. Quantitative research has found that 53% of the Australian public do not believe antidepressants are helpful for a person with depression [50], and qualitative research has indicated that even those supporting the use of medication may only see it as being acceptable for short-term use [51].

The current qualitative findings indicate that concerns about others' responses regarding professional help-seeking may play a crucial role in determining help-seeking behaviour. Very little quantitative research has investigated the effect of perceived stigma on help-seeking behaviour, however, Dew et al. [52] concluded that the responses of informal sources have a considerable impact. It has been suggested that when people reveal their depression to people who are highly qualified, others may infer that the help-seeker has a poor ability to cope [17]. Therefore, fears regarding help-seeking from professionals, and psychiatrists in particular, may be due to the desire of individuals to project an image of coping. According to evidence that people in the community consider support from the lay system rather than mental health professionals as being appropriate for depression [50,53,54], negative responses among the general public to professional treatment may be common.

It is also apparent from the current findings that people with depression have concerns that professionals themselves – both general practitioners and mental health specialists – may respond negatively to them. Expectations of such responses appear to be widespread amongst the general community, with both qualitative and quantitative research indicating that people expect general practitioners will be intolerant toward patients with emotional or depressive symptoms [55,56]. Furthermore, modifying health care providers' attitudes has previously been identified as a priority among people with depression [57]. Unfortunately, there is a lack of literature to support or refute the role of stigma in this context.

The current findings suggest that people may be most concerned about responses from mental health professionals. It is not clear if such worries are justified because research investigating responses of professionals has yielded mixed findings. A study from Switzerland found that mental health professionals, and psychiatrists in particular, hold negative stereotypes regarding people with mental illness [58], and Australian findings by Caldwell et al. [59] found that psychiatrists and psychologists hold more negative views than do other professionals about outcomes for people with depression. However, another Australian study by Griffiths et al [60] found lower stigma among those providing treatment or services for people with depression. Overall, it appears that we need to further consider and possibly address negative responses in professionals.

### Limitations

Two main limitations exist. Firstly, participants were self-selected, and therefore, the views of the group may not be representative of the wider community of people with depression. The representativeness of people who respond to advertisements requesting study participants is not known, but it is foreseeable that there may be differences between such people and others in the community with depression. One probable difference is the degree of willingness to discuss experiences of depression in a group setting. Whilst this tendency operates favourably in terms of disclosure yielding valuable information, it may also limit generalisability of the findings. Participants may also differ in other facets such as degree and chronicity of depression and social support. Although no such evidence is available in relation to depression groups, Grande et al [61] found that people participating in cancer support groups were more likely than non-participants from a cancer registry to be distressed and anxious, be without a partner and to report less support from a special person. Certainly people whose depression has (or has had) greater impact on their lives may have stronger views about the need for relevant studies and may be more willing to be involved in such research. Moreover, the low participation rate of young people and the high education level of many participants limit the generalisability of the findings. We also acknowledge that the overall sample size in the present study was modest, although we do not consider this a strong threat to the validity of the findings given that the number of participants was determined by the point at which saturation of views was attained. Further research using representative sampling and quantitative methodology may help to resolve the question of representativeness. Nonetheless, for the purpose of the current study (exploring the experiential domain), a sampling of the domain of responses rather than absolute representativeness is considered to have utility.

Secondly, findings may be limited by the accuracy of participant reports. In particular, perceived stigma may or may not accurately reflect the actual responses or attitudes held by other people. Overestimation of stigma regarding depression is a possibility given that there may be a tendency for people with depression to falsely perceive the reactions towards them by others as negative [62].

Despite these limitations, the current findings provide a valuable insight into the stigmatising dimensions of depression, highlighting areas where people may not have an adequate understanding about depression, and providing direction for the development of interventions to reduce stigma about depression.

## Conclusion

It is apparent that stigma can be a substantial problem for people with depression and may inhibit help-seeking from both informal and professional sources. There is clearly a need to address the stigma of depression, and this may involve both changing beliefs of the general public and challenging the perceptions of people with depression. Further research, particularly quantitative research, is required to provide input into the development of appropriate interventions for stigma. Nonetheless, the current findings suggest that public health programs should be focused on reducing stigma regarding the dimensions of *responsibility*, *repellence*, and *threat*. A strategy that replaces blame might involve providing a causal explanation for depression that incorporates sociological and biological components and is understandable by the lay public. Since there appears to be an association between 'mental illness' and dangerousness, it may be best to avoid the term 'mental illness' in labelling depression. Furthermore, it is important to explore methods that reduce feelings of discomfort among both sufferers and potential supporters of people with depression to avoid withdrawal and avoidance reactions. Although, for reasons other than destigmatisation, there is a need to address beliefs relating to the dimensions of *course *and *difficulties*, it is important to be sensitive to the complexity of intervening in these dimensions, and the possibility that improving such awareness may create stigma where there was none before. Additionally, we need to address people's reservations about help-seeking for depression, both from informal and professional sources, and modify the beliefs and responses of potential help-sources. An approach which provides people with an accurate view of depression but which emphasises the positive actions that can be sought and provided may prove useful.

## Competing interests

The authors declare that they have no competing interests.

## Authors' contributions

LB co-designed and conducted the study, carried out the data analysis and drafted the manuscript. KG, HC and AJ assisted in the design of the study and preparation of the manuscript. All authors have read and approved the final manuscript.

## Pre-publication history

The pre-publication history for this paper can be accessed here:


